# Problem nutrients in diet of under-five children and district food security status: Linear programming analyses of 37 stunting priority districts in Indonesia

**DOI:** 10.1371/journal.pone.0314552

**Published:** 2024-12-19

**Authors:** Umi Fahmida, Indriya Laras Pramesthi, Sari Kusuma, Arienta R. P. Sudibya, Rahmawati Rahmawati, Dini Suciyanti, Gusnedi Gusnedi, Aly Diana

**Affiliations:** 1 Southeast Asian Ministers of Education Organization Regional Centre for Food and Nutrition (SEAMEO RECFON)–Pusat Kajian Gizi Regional (PKGR) Universitas Indonesia, East Jakarta, Indonesia; 2 Department of Nutrition, Faculty of Medicine, Universitas Indonesia − Dr. Cipto Mangunkusumo General Hospital, Jakarta, Indonesia; 3 Department of Nutrition, Health Ministry Polytechnic of Padang, West Sumatra, Indonesia; 4 Department of Public Health, Faculty of Medicine, Universitas Padjadjaran, Padjadjaran, Indonesia; University of British Columbia, CANADA

## Abstract

**Background:**

In Indonesia, food security and dietary patterns varied by regions. This might lead to differences in problem nutrients (PN) and should be considered in developing local-specific food-based recommendations (FBRs) for stunting prevention.

**Objectives:**

This study aims to identify PNs in diet of under-five children in selected 37 stunting priority districts in Indonesia and assess whether the number of PNs was associated with district food security status.

**Methods:**

Linear programming analysis (LP) using Optifood was done using single 24-hour dietary recall data Ministry of Health 2016 Food Consumption Survey. PN was defined as nutrient which did not meet 100% Recommended Nutrient Intake (RNI) based on Indonesian-RNI in the 2-best-diets scenario. District’s food security status was determined using Food Security Vulnerability Atlas 2018.

**Results:**

The top three PNs amongst under-five childrenwere iron, folate, vitamin B12 (6-11mo); folate, calcium, zinc (12-23mo); folate, calcium, vitamin C (24-35mo); and calcium, folate, vitamin C (36-59mo). There were no significant differences in number of PNs based on food security status. After integrating the nutrient-dense foods into FBRs, the number of districts with dietary inadequacy decreased for the top-3 problem nutrients in each age group, with the exception of iron for infants.

**Conclusions:**

Our findings showed that problem nutrients and dietary inadequacy were prevalent in diet of under-five children, even in food-secure areas. Promoting locally available nutrient-dense foods through FBRs (nutrition-specific intervention) and ensuring availability and access to these foods (nutrition-sensitive intervention) are recommended. Additional intervention(s), particularly for iron in infants, are required.

## Introduction

Indonesia still faces major challenges in alleviating malnutrition, especially among under-five children. Stunting is the common nutrition problems among this age group, which are closely related to inadequate nutrient intake from the diet. This situation has not improved significantly over the last decade, with stunting amongst under-five children being high i.e. 37.2% in 2013 [1] and 30.8% in 2018 [2]. Linear programming analysis of 2010 national dietary survey data amongst under-two children and two studies in sub-population groups of under-two children revealed that iron, zinc, calcium, niacin and folate are typical problem nutrients (PNs) in complementary feeding diets of young children in Indonesia [3].

The use of suitable locally-available foods has been emphasized by the World Health Organization (WHO) and UNICEF in the Global Strategy for Infant and Young Child Feeding [4] as well as the Food-based Dietary Guideline (FBDGs) in many countries. However, despite the potential for locally-available nutrient-dense foods to increase dietary adequacy, their usage in the nutrition-specific program must still be optimized.

WHO has provided “Optifood”, a software that uses Linear Programming (LP) to formulate food-based recommendations (FBRs) that is locally specific and affordable.These FBRs which take into account cultural diversity and differences in food availability are more likely to result in long-term improvements in dietary practices than general recommendations. On the other hand, food security can affect diet quality and therefore may influence the number and type of problem nutrients in the diet [5–7]. Dietary pattern also changes along the childhood period from infancy, complementary feeding period, and later period. Arecent study found that dietary quality declines with age and may begin as early as 1 year old [8], or even sooner [9].

Understanding whether number and type of PNs are affected by age and food security at the district level is critical for developiong a more effective approach for implementing nutrition-specific and nutrition-sensitive interventions to reduce stunting. This information is especially important in districts with a high prevalence and number of stunted children are high (the stunting priority districts) which have grown from 8 districts in 2017, to 100 districts in 2018, 160 districts in 2019, and eventually cover 514 districts in Indonesia in 2020–2024 [10]. The aim of this study is to identify PNs in the diet of under-five children in the 37 stunting priority districts in Indonesia and determine whether the number of PNs were associated with age of the children and the district’s food security status.

## Methods

This study used secondary data from the Ministry of Health of Indonesia’s 2016 National Nutrition Consumption Monitoring Survey (*Survei Pemantauan Konsumsi Gizi*) for under-five children. The sample was randomly selected from households with children under the age offive, using Probability Proportion to Size (PPS). Dietary intake data was collected using a single 24-hour dietary recall.

Data of 11,040 children aged 6–59 from 37 selected districts in 33 provinces in Indonesia were checked for completeness. Children with missing data on dietary intake, age, or breastfeeding status were excluded from the analysis. Out of 37 selected districts, 32 districts were selected based on the following criteria: (1) the districts were included in the list of 160 stunting priority districts according to the Government of Indonesia stunting reduction program in 2019; (2) the districts had the highest stunting prevalence within each province; and (3) the local specific FBRs of the districts has not been developed. Pasrticularly for East Java Province, five districts were selected based on those three criteria and represented different geographic typologies i.e. highlands, lowlands, and coastline.

### Food composition tables

Food nutrient values were obtained from the Optifood internal reference food composition tables, which contains nutrient composition data for approximately 2000 foods. Nutrient values for any food items not available in the Optifood food composition tables were compiled using the Indonesian food composition tables developed for the SMILING Project [11], the FAO/INFOODS Food Composition Databases for Asia [12], augmented where necessary with nutrient values from the US Department of Agriculture [13] and ProPan 2.0 [14]. The phytate content of foods, including adjustments for fermentation, was compiled from Global Food Composition Database for Phytate version 1.0 [15]. Nutrient contents of raw foods in the food composition table, when consumed in the cooked state, were adjusted for nutrient losses during cooking using retention factors [16]. Nutrient values for all wheat flour products were adjusted to reflect the Indonesian government mandatory fortification levels (thiamine 2.5 mg/kg, riboflavin 4 mg/kg, iron 50 mg/kg, and zinc 30 mg/kg) [17]. Nutritional information from commercially available foods was obtained to account for fortification.

### Linear programming analysis

The LP analysis was performed using linear optimization (Excel’s solver function) in Microsoft Excel software or WHO Optifood V4.0.14.0 (June 16^th^, 2015). LP-Optifood analysis was done in several steps as follows: 1. preparing the list of foods consumed by subjects to be entered into Optifood software; 2. preparing the list of food groups and sub-groups, including the frequency of consumption; 3. performing the "check diet" for feasibility analysis of the FBR; 4. analysing the data to get 2-best diets (optimal-case-scenario) which was closest to existing food pattern i.e. median weekly frequency of food groups (Best Diet FP) and which may deviate from the existing food pattern (Best Diet No-FP), the worst-case (%RNI when each nutrient was minimized) dan the best-case (%RNI when each nutrient was maximized).Based on Best Diet No-FP, Best-case and Worst-case each nutrient was then categorized as either absolute problem nutrient (PN_a_), partiap problem nutrient (PN_p_), dietary inadequate (DI) or dietary adequate (**Table 1**); 6. identifying the proposed nutrient-dense foods of sub-groups to fill the nutrient gaps; 7. determining the most optimal FBRs by comparing several FBR alternatives. For the nutritional adequacy target, the Indonesian Recommended Nutrient Intake (*Angka Kecukupan Gizi*) 2019 [18] was used as reference (**Table 2**).

**Table 1:** Criteria used in LP analysis for defining problem nutrients and dietary inadequacy, based on %RNI.

Category of nutrient	Percentage of the Indonesian RNI		
	Best-diet No-FP	Best-case	Worst-case
Dietary adequacy	≥100%	≥100%	≥65%
Dietary inadequacy	≥100%	≥100%	<65%
Problem nutrient, partial	<100%	≥100%	<65%
Problem nutrient, absolute	<100%	<100%	<65%

**Table 2:** Nutrient recommendation based on Indonesian RNI^1^ used in the LP analyses.

Nutrient	Unit	6–11 months	1–3 years	4–6 years
Energy	kcal/day	800	1350	1400
Protein	gram/day	15	20	25
Fat	%energy	40	30	32
Vit A	mg/day	400	400	450
Thiamine	mg/day	0.3	0.5	0.6
Riboflavin	mg/day	0.4	0.5	0.6
Niacin	mg/day	4	6	8
Vit B6	mg/day	0.3	0.5	0.6
Folate	mcg/day	80	160	340
Vit B12	mg/day	1.5	1.5	1.5
Vit C	mg/day	50	40	45
Iron	mg/day	11	7	10
Zinc	mg/day	3	3	5
Calcium	mg/day	270	650	1000

^1^Source: *Angka Kecukupan Gizi* 2019

### Categorization of problem nutrient and food security status

To identify if district had more than one PN in each age group, we sum the number of PNs in all four age groups. Districts having five or more PNs were categorized as ‘high PN’. The district’s food security was categorized using the Food Security Vulnerability Atlas (FSVA) which was published in 2018 [19].

The FSVA includes 13 indicators based on a review of data availability at the district level and their ability to measure various aspects of food and nutrition security. The FSVA divides these indicators into two sets: chronic food and nutrition insecurity and transitory food insecurity. The chronic food and nutrition security includes indicators on food availability (i.e. ratio of per capita cereal consumption to per capita production), food access (i.e. villages in each district that have access to roads passable by four-wheeled vehicle or to waterways passable by boat throughout the year, households’ access to electricity, population living below the national poverty line), and food utilization (i.e. villages with health facilities more than 5 kilometres away, households without access to clean drinking water at a distance of more than 10 meters from a septic tank, illiteracy rate among female ages 15 and above, prevalence of stunting among children under five years of age, life expectancy). The transitory food insecurity indicators describe climatic and environmental factors (i.e. number of natural disasters with potential impact on food access and utilization from 2000–2013, rainfall variability, district classification of negative monthly rainfall change based on strength of El Niño-Southern Oscillation (ENSO) signal, average rice production loss due to drought from 1990–2013, deforestation that affect food insecurity from an availability and access perspective. The nine indicators associated with chronic food insecurity were integrated into a single composite indicator to describe the overall district food security classification. A composite indicator representing the overall food and nutrition security situation was constructed and used to classify districts into six priority groups. Districts in priority groups 1 and 2 are relatively more vulnerable to food and nutrition insecurity; those in priority groups 3 and 4 are moderately vulnerable; and those in priority groups 5 and 6 are less vulnerable or food secure. In our analyses, districts were categorized as food insecure if the FSVA priorities were 1–3 (severe, moderate and mild food insecurity) and food secure if FSVA priorities were 4–6 (low, middle and high food secure) [7].

The ethical approval for the study was obtained from Ethical Committee, Faculty of Medicine Universitas Indonesia No. KET.46/UN2.F1/ETIK/PPM.00.02/2020.

### Statistical analysis

Statistical analysis was completed with Stata 14 (StataCorp LLC, Texas, USA). Descriptive statistics were calculated for all variables. Independent t-test was conducted to assess the association between food security status and number of PNs, p < 0.05 level of significance was used.

## Results

In total, dietary intake data from 9,847 under-five children were included in LP analysis. Number of dietary data varied with a range of 92 to 308 under-five children per district (average 266 children). The number of food items included in LP analyses varied greatly based on the dietary intake data. The minimum number of food items was 16 amongst 6-11mo infants in Nunukan (North Kalimantan) and the maximum number was 130 amongst 36-59mo children in Bantul (DI Yogyakarta), **Table 3.**

**Table 3:** Number of food items identified measure by a single 24 hour dietary recall for LP analyses, by district and age group.

No	Province	District	Number of Variety of Foods (N)				
			Total per District	6–11 mo	12–23 mo	24–35 mo	36–59 mo
1	Aceh	Aceh Timur	110	37	53	69	75
2	Sumatera Utara	Simalungun	127	29	84	78	93
3	Sumatera Barat	Solok	146	35	97	100	126
4	Jambi	Kerinci	85	30	46	55	63
5	Riau	Kampar	137	39	89	81	108
6	Sumatera Selatan	Muara Enim	106	31	67	59	93
7	Bengkulu	Bengkulu Utara	118	44	85	68	96
8	Lampung	Tanggamus	140	45	84	86	112
9	Kepulauan Bangka Belitung	Bangka	129	47	73	71	103
10	Kepulauan Riau	Lingga	118	37	77	92	86
11	Kalimantan Barat	Sintang	121	32	74	83	84
12	Kalimantan Tengah	Kapuas	76	28	44	44	57
13	Kalimantan Selatan	Tanah Bumbu	117	21	58	77	97
14	Kalimantan Timur	Kutai Barat	94	22	62	50	80
15	Kalimantan Utara	Nunukan	79	16	39	39	57
16	Banten	Lebak	146	47	100	93	110
17	Jawa Barat	Majalengka	149	49	92	112	121
18	Jawa Tengah	Pekalongan	126	47	92	99	94
19	DIY	Bantul	171	38	115	111	130
20	Jawa Timur	Kediri	137	44	71	96	101
21		Jember	112	34	68	78	76
22		Bondowoso	111	41	81	68	73
23		Nganjuk	125	40	60	79	91
24		Sampang	127	40	83	94	96
25	Bali	Buleleng	151	41	93	88	112
26	NTB	Bima	127	33	79	88	108
27	NTT	Manggarai Barat	87	21	61	63	73
28	Sulawesi Utara	Bolaang Mongondow	90	30	51	60	62
29	Sulawesi Tengah	Parigi Moutong	102	27	68	68	78
30	Sulawesi Selatan	Bone	135	32	79	95	101
31	Sulawesi Tenggara	Kolaka	110	40	69	70	84
32	Gorontalo	Pohuwato	78	25	57	54	57
33	Sulawesi Barat	Mamasa	119	35	69	79	98
34	Maluku	Kepulauan Aru	95	34	61	54	68
35	Maluku Utara	Kepulauan Sula	83	23	46	53	59
36	Papua Barat	Manokwari	115	45	75	80	91
37	Papua	Mamberamo Tengah	36	19	21	26	33

**Fig 1:**
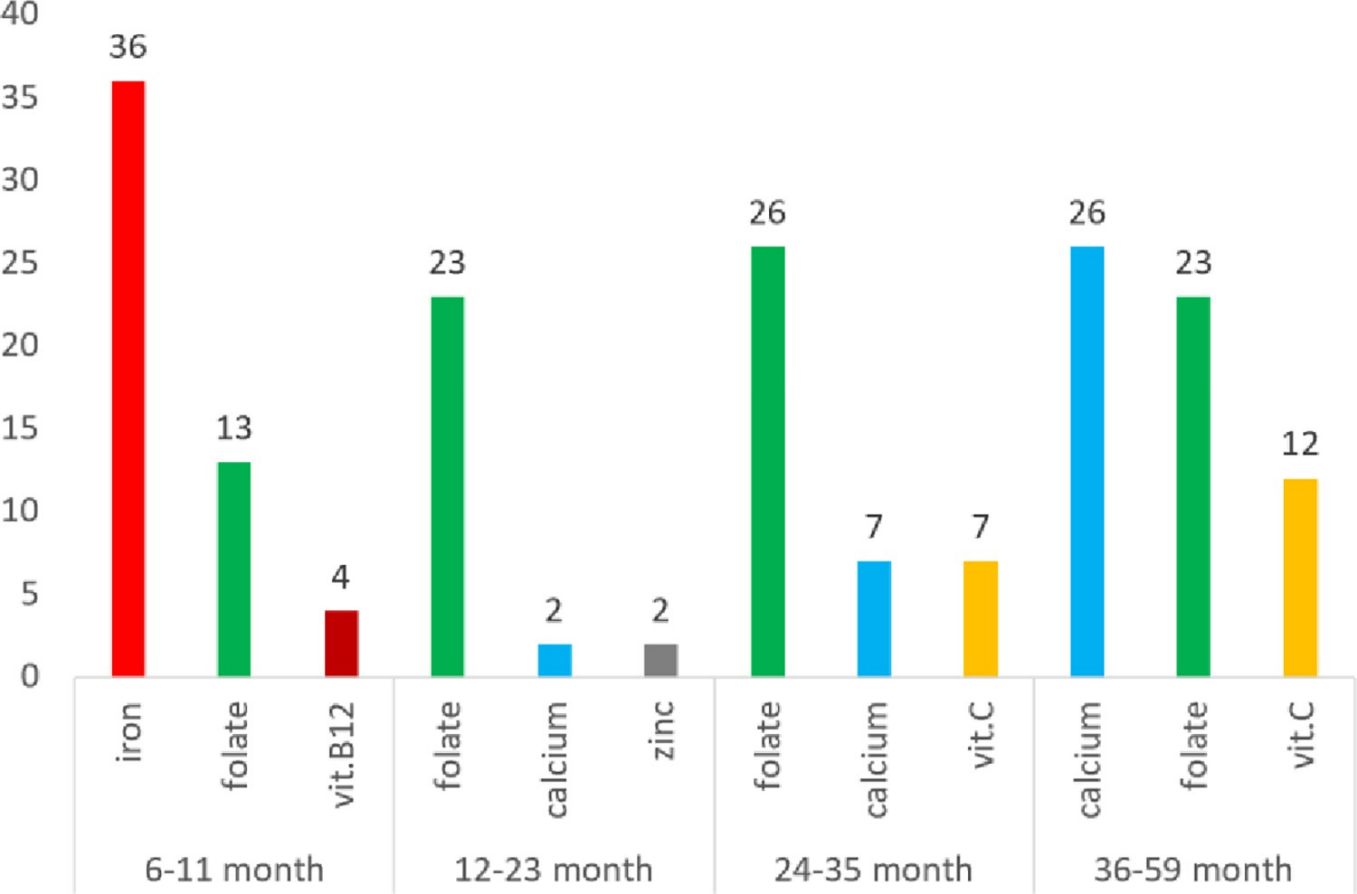
Top 3 problem nutrients in the 37 stunting priority districts, by age group.

Majority of the 37 districts had partial or absolute PNs in their food pattern. Infants (6-11mo) had the largest percentage of districts with at least one PN (35/37 = 95%). While the proportions of district with PNs decreased in the second year of life (25/37 = 68%), the proportion increased toward the end of the under-five year period (28/37 = 76% in 24-35mo and 33/37 = 89% in 36-59mo children). The top-3 PNs in each age group were: iron, folate, vitamin B12 (6-11mo); folate, calcium, zinc (12-23mo); folate, calcium, vitamin C (24-35mo); and calcium, folate, vitamin C (36-59mo), **Fig 1**. Besides the PNs, some nutrients were not adequate (dietary inadequate) as its minimized RNI is still below the EAR (<65% RNI). On average, most districts had 1–2 PNs and additional 1–3 dietary inadequacy (**Tables 4 and 5**).

**Table 4:** Identified problem nutrients and dietary inadequacy in 6-23mo children, by district.

Kabupaten	6–11 months											12–23 months										
	Ca	Fe	Zn	Vit C	Thiamin	Ribofv	Niacin	Vit B6	Folate	Vit B12	Vit A	Ca	Fe	Zn	Vit C	Thiamin	Ribofv	Niacin	Vit B6	Folate	Vit B12	Vit A
Aceh Timur		PNa*		DI			PNa	DI	PNa			PNa								PNa*		
Simalungun		PNa*		PNp			DI		PNa			DI	DI		DI			DI	DI*	PNa*	DI	
Solok		PNa*	PNa	PNa					DI	DI		PNa	PNp						PNa	PNa*		
Kerinci		PNa*		DI		DI	DI	DI	DI*	DI			DI	DI	DI				DI	PNa	DI	
Kampar		PNa*						DI	PNa	DI			DI	PN*	DI		DI		DI	PN*	DI	
Muara Enim		PNa*	DI	DI			DI	DI	DI	DI									DI*	PNa*		
Bengkulu Utara		PNa*						DI	DI	DI*			DI	DI	DI		DI	DI	DI	DI*	DI	
Tanggamus		PNa*						DI	PNp	DI				PNa*						DI		
Bangka		PNa*						DI	DI	DI		DI	DI		DI			DI	DI	PNa*		
Lingga		DI	DI	DI			DI	DI	DI	DI									DI	DI	DI	
Sintang		PNa*	DI	DI		DI	DI*	DI	DI	DI		PNp	DI		DI			DI	DI*	PNp*	DI*	
Kapuas		PNa*	DI	DI				DI*	PNa*	DI										PNa*		
Tanah Bumbu		PNa*	DI				DI	DI	PNp	DI*										PNa*		
Kutai Barat		PNa*		DI			DI	DI	PNa*	PNa*									DI	PNa	DI*	
Nunukan		PNa*						DI	PNa	DI										PNa		
Lebak		PNa*	DI	DI	DI		DI	DI	DI	PNa*		PNp			PNp					PNa		
Majalengka		PNa*																		DI		
Pekalongan		PNa*	DI	DI			DI	DI	PN	DI*		DI	DI		DI				DI	DI*	DI*	
Bantul		PNa*					DI	DI	DI	DI*		DI		DI	DI			DI	DI	DI*	DI	
Kediri		DI*						DI	DI	DI										DI		
Jember		PNa*	DI	DI	DI		PNa	PNa	PNp	DI										PNa		
Bondowoso		PNa*						DI	PNa*										DI	PNa*	DI	
Nganjuk		PNa*	DI	DI			DI	DI	DI	DI*		DI*	DI*	DI*	DI		DI	DI*	DI*	DI*	DI*	
Sampang		PNa*						DI	PNa*											DI*		
Buleleng		PNa*	DI	DI			DI	DI	DI	PNa*		DI								DI		
Bima		PNa*							Pna	PNa*										PNa		
Manggarai Barat		PNa*					DI	DI	DI				DI	DI	DI		DI	DI	DI	PNa*	DI	
Bolaang Mongondow		PNa*	DI				DI	DI	PNa*	DI										PNa*		
Parigi Moutong		PNa*						DI	DI	DI		DI	DI	DI	DI				DI*	DI*	DI	
Bone		PNa							DI											PNa		
Kolaka		PNa*	DI	DI			DI	DI	PNa	DI										PNa		
Pohuwato		PNa	DI	DI	DI		DI	DI	DI	DI								DI	DI	PNp*	DI	
Mamasa		PNa*						DI	DI	DI		DI	DI	DI	DI		DI	DI	DI	DI	DI	
Kepulauan Aru		PNa*						DI	PNa	DI									DI	PNa*	DI	
Kepulauan Sula		PNa*	DI	DI			DI	DI	PNa	DI*					DI					PNa*	DI	
Manokwari		PNa*	DI					DI	DI	DI										DI		
Mamberamo Tengah		PNa*	DI	DI			DI	DI	DI	DI										PNa*		
Number of problem nutrients	0	35	1	1	0	0	2	1	13	4	0	4	1	2	1	0	0	0	1	24	0	0
Ranking		1st							2nd	3rd		2nd		3rd						1st		

PNa = absolute problem nutrient, PNp = partial problem nutrient, DI = dietary inadequacy

**Table 5:** Identified problem nutrients and dietary inadequacy in 24-59mo children, by district.

Kabupaten	24–35 months											36–59 months										
	Ca	Fe	Zn	Vit C	Thia min	Ribofv	Niacin	Vit B6	Folate	Vit B12	Vit A	Ca	Fe	Zn	Vit C	Thia min	Ribofv	Niacin	Vit B6	Folate	Vit B12	Vit A
Aceh Timur	PNa			PNa*		DI*			PNa*	DI*	DI	PNa*	PNa	PNa	PNa*				PNa	PNa*		PNa*
Simalungun	PNa			PNa					PNa*			PNa*		PNa*			DI*	DI	DI	PNa	DI	DI
Solok	PNa			DI					PNa*		DI	PN*	DI	DI	PN*		DI	DI	DI	PN	DI	DI
Kerinci									PNa*			PNa*		PNa	DI			DI	DI	PNa*	DI	DI
Kampar	PNa*		PNa*	DI					PNa*			PNa*	PNa*		PNa*			DI		PNa*	DI	DI
Muara Enim				DI*					PNa*			PNa*	DI		DI			DI		PNp*	DI	
Bengkulu Utara				DI					PNa*			PNa*		DI*	DI*					PNa*		
Tanggamus	PNp*		PNa*	DI		DI		DI	DI*	DI	DI	PNa*			PNa*					PNp		DI
Bangka	DI	DI		DI				DI	DI		DI	DI	DI	DI	DI	DI	DI	DI	DI	DI	DI	DI
Lingga	DI		DI	DI		DI	DI	DI	DI	DI	DI	PNp*	DI	DI*	DI*		DI	DI*	DI	DI	DI	DI
Sintang				DI					PNa*						DI*					PNp*		DI
Kapuas	DI			DI*					PNa*		DI	DI			DI*		DI			PNa*	DI	DI
Tanah Bumbu	DI			DI		DI			DI	DI	DI	PNp*	DI	DI	DI		DI			PNa*	DI	DI*
Kutai Barat		DI		DI		DI	DI	DI	PNa*	DI	DI	PNp			DI					PNa		
Nunukan									PNa			DI	DI	DI	DI		DI		DI	PNa*	DI	DI
Lebak				DI*					PNa*			PNa	DI*	DI*	DI*		DI*		DI*	DI*	DI*	DI*
Majalengka	DI*	DI		DI	DI	DI	DI	DI	DI*	DI	DI	DI*	DI*	DI*	DI	DI	DI	DI*	DI*	DI*	DI*	DI
Pekalongan	DI		DI	DI		DI	DI	DI	DI*	DI	DI	PNa	DI	DI	DI	DI	DI	DI	DI	PNa	DI	DI
Bantul	DI		DI	DI		DI	DI	DI	DI	DI	DI		DI	DI	DI*	DI	DI	DI	DI	DI*	DI	
Kediri									PNa*			PNa*	DI	DI*	DI		DI	DI	DI	PNa*	DI	DI
Jember	DI			DI				DI	PNa*	DI	DI	PNa*			DI*					PNa*		DI
Bondowoso	PNa			PNa*					PNa*		DI	PNa*	DI	DI*	PNa*				DI	PNa*	DI	DI
Nganjuk	DI*	DI	DI	DI	DI		DI		PNa*	DI	DI	PNa*	DI	DI*	DI		DI	DI	DI	PNa*	DI	DI
Sampang	DI*			PNa*					PNa*	DI*	DI*	PNa*			PNa*					PNa*		DI*
Buleleng	DI			DI					PNa	DI	DI	PNp	DI	DI	DI		DI	DI	DI	DI	DI	DI
Bima	DI			PNa*					DI*	DI	DI	PNa			PNa*					DI		DI
Manggarai Barat			DI	DI		DI		DI	PNa*	DI	DI	PNa*	DI	DI*	DI*		DI	DI*	DI	PNa*	DI*	DI*
Bolaang Mongondow				DI*					PNa*			PNa*		PNa*	PNa*			DI*		DI*	DI	DI
Parigi Moutong				DI					DI*		DI	PNa*		PNp	PNa*					PNa*		PNa
Bone	DI	DI	DI	DI		DI	DI	DI	DI*	DI	DI	DI*	DI	DI	DI	DI	DI	DI	DI	DI*	DI	DI
Kolaka				DI		DI			PNa	DI	DI	PNa		DI	PNa		DI			PNa	DI	DI
Pohuwato				DI				DI	PNa	DI	DI	PNa*		DI	PNa*			DI	DI	PNa*	DI	DI
Mamasa	DI*			DI		DI			DI	DI	DI	PNa		DI	DI		DI			DI		DI
Kepulauan Aru	PNa		DI	PNp		DI			PNa*	DI	DI	PNa*		DI	PNp					PNa*	DI	DI
Kepulauan Sula				PNa		DI			PNa*			PNa*	DI	DI*	PNa*		DI*	DI	DI	PNa*	DI	DI
Manokwari	DI	DI	DI	DI		DI	DI	DI	PNa*	DI	DI	Pna*	DI	PNp*	DI	DI	DI*	DI	DI	PNp*	DI	DI
Mamberamo Tengah									PNa*	PNa		PNa		PNa*						PNa*	DI	DI
Number of problem nutrients	7	0	2	7	0	0	0	0	26	1	0	29	2	7	12	0	0	0	1	26	0	2
Ranking	2nd			3rd					1st			1st			3rd					2nd		

PNa = absolute problem nutrient, PNp = partial problem nutrient, DI = dietary inadequacy

**Fig 2:**
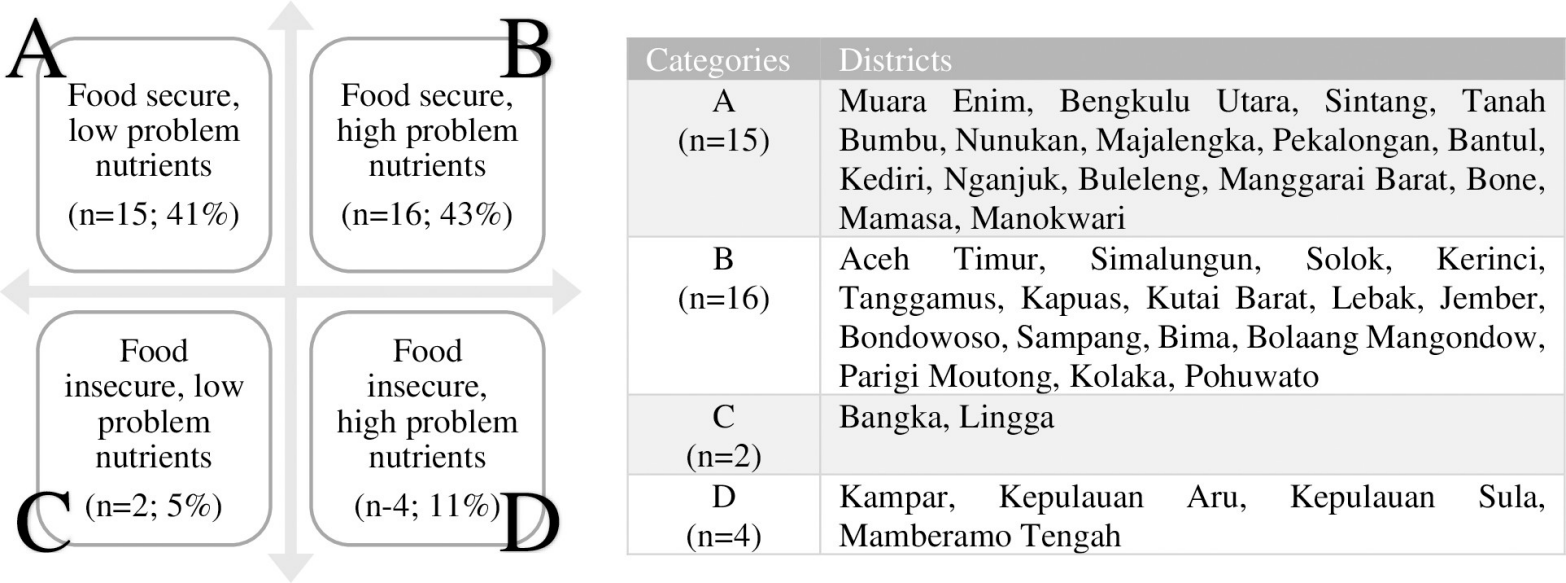
Distribution of 37 stunting priority districts by food security status and number of problem nutrients in under-five children. • Categorization of food security status based on Food Security Vulnerability Atlas (FSVA) 2018: 1 = severe food insecure; 2 = moderate food insecure; 3 = mild food insecure; 4 = low food secure; 5 = middle food secure; 6 = high food secure, • Categorization of problem nutrient, based in total number of PN in all four age groups with median [6] as the cut-off, low PN< 6 problem nutrients; high PN ≥6 problem nutrients.

Out of the 37 districts, 31 districts (84%) were categorized as food secure and 6 districts (16%) as food insecure based on FSVA priorities. Nineteen districts (51%) had at least 6 PNs in all the four age groups based on LP analyses. Based on combined categories of food security and number of PNs, almost half of districts (43%) were food secure but had at least 6 PNs (**Fig 2**). The independent t-test between number of PNs and food security status was non-significant (p = 0.501). The number of nutrients which were adequate varied by age group, with the least number in the 35-59mo age group (0–1 nutrients only) followed by 24-35mo (1 nutrient), 12-23mo (2 nutrients), and 6-11mo (3 nutrients).

**Fig 3:**
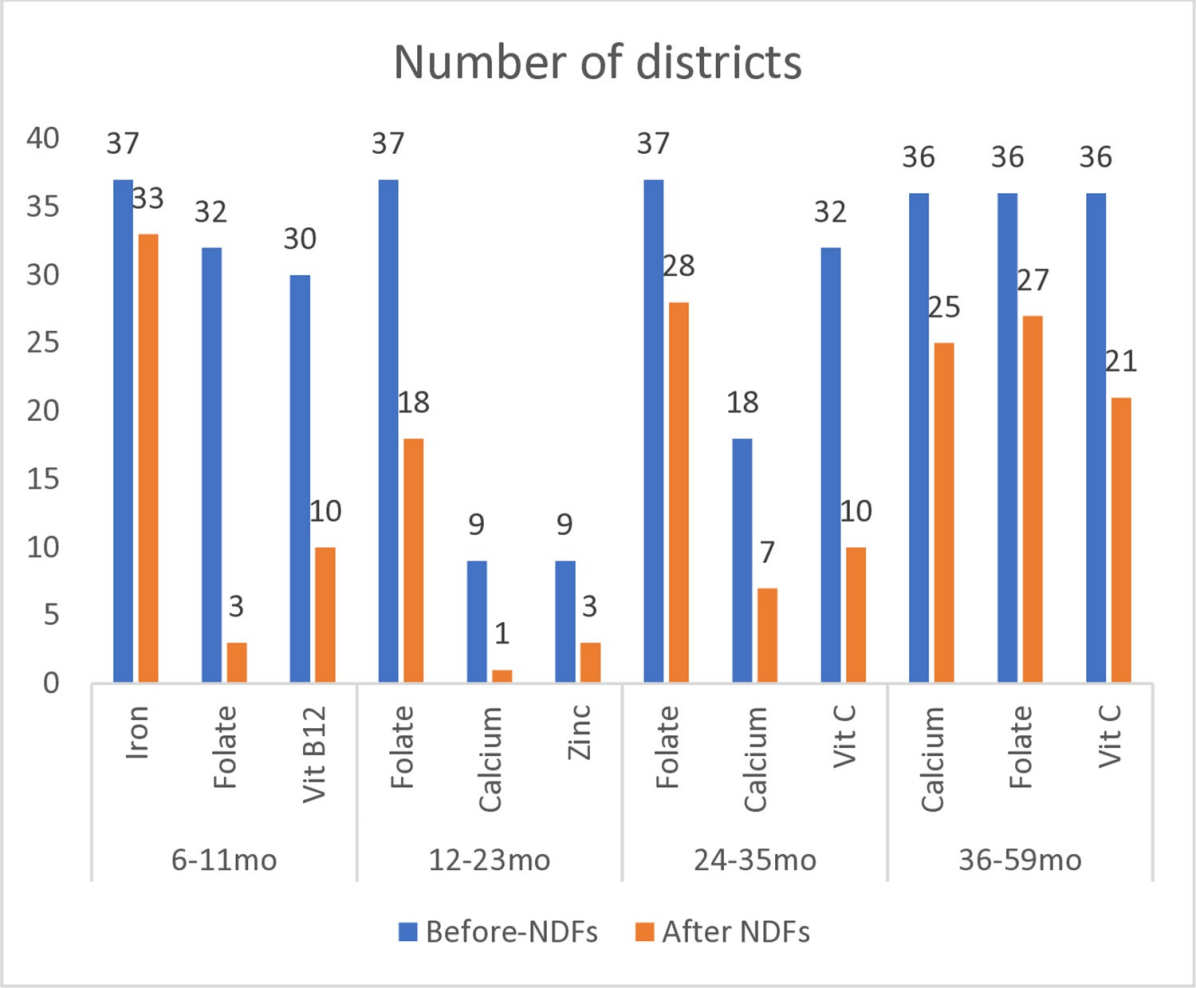
Number of districts with dietary inadequacy before and after nutrient-dense foods (NDFs) were included in the diet, based on LP analysis in Module 3 (worst-case scenario).

Nutrient-dense foods with potential to improve dietary adequacy of the problem nutrients were identified from LP analyses and were included in the final FBRs (**S1–S5 Tables**) After incorporating these nutrient-dense foods in the FBRs, the number of districts with dietary inadequacy decreased for the top-3 problem nutrients in each age group, with the exception of iron for infants (**Fig 3**).

## Discussion

This study is the first study to identify problem nutrients of under 5-years-old children using national data from 37 stunting priority districts in Indonesia. Among the top 3 PNs, folate was the PNs in all age groups, iron was considered problem in almost all districts at earlier age (6-11mo), while calcium was considered problem at later age (11mo and older). However, the number of PNs was not associated with the district’s food security status.

Number of districts with PNs was highest among youngest infants aged 6-11mo. This finding was consistent with our prior analysis using the 2013 Indonesian Basic Health Survey data, which revealed that children 6-11mo had four PNs (iron, zinc, calcium, niacin) as compared to no PN in the older children aged 12-23mo [3]. However, in these 37 stunting priority districts, we also found that older children above 24mo had more PNs than their 12-23mo counterparts, despite more variety of foods they consumed [20].

The fact that all districts had PN(s) and/or dietary inadequacy in their food patterns suggests that feeding practices needs to be improved, particularly in terms of intakes of animal source foods, fruits, and vegetables which are rich in iron, calcium, zinc, B12, folate, and vitamin C. Recent review of 23 studies employing diet modelling on children aged 6-23mo found that the diets are inadequate in iron, zinc, and calcium. The review also found that requirements for vitamin A, thiamine, riboflavin, niacin, folate, and vitamin C were not always met [21]. Previous study reported that consumption of plant based foods (e.g. rice, maize, legumes), fibre, and polyunsaturated fat, which was common among children aged 6–24 months old, was inversely associated with nutrient density of the complementary diet for vitamin C, calcium, and iron [22].

Unexpectedly, folate was a PN in all age groups, a finding which was not reported in previous study that used a nationally representative data from Indonesia [3]. Folate is naturally present in a wide variety of foods, including vegetables (especially dark green leafy vegetables), fruits and fruit juices, nuts, beans, eggs, and rice [13, 23], which are commonly found in almost all areas in Indonesia. The finding suggests that consumption of fruits and vegetables is not yet optimal and was in line with national figure from Indonesian Basic Health Survey Report 2018 that over 90% of Indonesian population did not consume fruits and vegetables adequately in terms of frequency and portion [2].

Iron is the most common PN among infants aged 6–11 months, which confirmed findings in previous studies [3, 21]. It is known that iron concentrations in breastmilk decrease as infants grow older [24], therefore more iron from complementary feeding is required to meet the RNI. The challenge for 6–11 mo children to meet all of their nutrient requirements from locally available and typically unfortified foods has been primarily caused by predominantly cereal-based and monotonous diet, resulting in low dietary diversity, low nutrient density, and poor micronutrient bioavailability [25]. Although infants who consumed fortified infant products and fortified formula milk had significantly higher intakes of iron compared to infants who did not consume these products, intakes were still inadequate for iron, due to inadequate nutrient densities of fortified infant foods, small quantities consumed, and/or infrequent consumption of these products [9, 26].

Across all age groups, folate and calcium were the most problematic nutrients. Inadequate intake of folate during infancy and early childhood has negative consequence on brain development [27]. The absence of calcium as PN at 6-11mo is most likely due to breastmilk, which we included in the LP analyses in accordance with the public health message of breastfeeding on demand. According to the Indonesian Basic Health Survey 2018, the national rate of exclusive breastfeeding amongst infants under the age of 6 months was only 37.3% [2]. This suggests that dietary adequacy of calcium may have been over-estimated in our analyses and that calcium may still be PN given the actual rate of exclusive breastfeeding.

The LP analysis of these 37 districts revealed that zinc, a type-II nutrient associated with stunting, was not identified as PNs in most districts, a finding which was in contrast with previous study [21]. In our analysis, zinc was found as the PNs only in some districts, mainly amongst the 36-59mo children. This suggests that food basket and food pattern of these under-five children are generally sufficient to ensure adequacy of zinc intake. Fish was zinc-rich food which was commonly consumed in many districts in Indonesia and fish consumption has increased significantly due to promotion to consume fish conducted by Ministry of Fishery (the *Gemarikan* which means “Let’s Eat Fish” movement). In Indonesia, fish consumption rose from 38.14 kg/capita.year in 2014 to 55.95 kg/capita.year in 2019 [28].

Our finding was consistent with previous study that dietary quality starts to decline as early as one year old [3]. In younger age, children experienced variety of food for the first time. As children get older, they gain greater control over what they eat, which can have favorable or negative impact on dietary quality [29, 30]. This emphasizes the need of promoting healthy and balanced diet beyond the complementary feeding period. Nutrition education in early childhood education centres can be the potential channel to establish healthy eating habit amongst toddlers who begin to make dietary choices. One of the approaches can be to teach children to identify and categorize food which can be consumed sometimes (i.e., unhealthy) or anytime (i.e., healthy) as previous study found that pre-schoolers who were better at identifying food as healthy or unhealthy were more likely to select healthier option [22].

The finding that food insecurity had no significant influence on the number and type of PNs suggest that the problem was more with food consumption (i.e. less favourable feeding and dietary practices) rather than food availability. In addition, parents may compromise their food intake to protect their younger children from food shortage in the food insecure household and earlier study found that food security has linear relationship with diet quality in adults while pattern was inconsistent in children [20].

Most of the PNs found in these 37 districts were absolute PNs, which suggest limitation in the *current* food basket of the children. However, this does not necessarily mean that the community food basket is inadequate; rather, the foods with potential to increase of the PNs may be available locally but are not commonly fed to children. In our earlier study in East Lombok we promoted anchovy which was not traditionally included in children’s diet but was locally available and consumed by older family members. The result showed that introduction of anchovy powder can increase calcium intake which was the PN amongst the under-two-year-old children, suggesting that it is possible to introduce nutrient-dense food from the family food basket into the child’s food basket [11]. Furthermore, our current LP analysis has shown that the introduction of nutrient-dense foods using traditional recipes of composite dishes (dishes containing three or more food groups) have also been shown to meet the requirement of the PNs iron, folate, vitamin C in several districts across different regions/islands.

The LP analyses conducted in this study were the first to identify the PNs in 33 out of 34 provinces in Indonesia. LP analyses were also conducted by trained nutritionists who were familiar with the local food patterns and therefore could put the FBRs into a local context. The limitations of the study were the small sample size (<50) in some district-age groups, especially for the 6-11mo infants, which may limit the number of identified food items consumed. Nonetheless, we used the most recent national representative dataset, which can still be used to develop local specific FBRs.

Since the aim of LP analyses is optimization, not all FBRs can ensure dietary adequacy of all nutrients. Dietary adequacy was particularly difficult to meet for iron in 6-11mo infants; for folate in 12-23mo children and 24-35mo children; and for calcium and folate in 36-59mo children. LP analyses indicated that the gap for these nutrients in the worst-case scenario (minimized scenario) ranged between 30–40%RNI; or equivalent to nutrient gap of 25–35%RNI when compared with 65%RNI (≈EAR level). In our earlier study, we used shredded chicken liver, shredded fish, and anchovy powder to increase intakes of the PNs iron, zinc, and calcium [31]. We also found that mungbean was rich in folate. Using these nutrient-dense foods, the ‘nutrient gap’ identified in our analyses of these 37 districts can be achieved by providing shredded liver (*abon hati*) of 16–19 grams/day (for iron), dried anchovy powder of 14–17 grams/day (for calcium), or mung beans of 22–27 grams/day (for folate), all ofwhich are feasible portions for children. Several nutrient-dense foods identified from the LP analyses in the current study can be promoted further to increase intakes of the typical PNs amongst the under-five children. These include fish, seaweed, sugar-palm fruit, and mung beans; as well as local recipes in form of composite dishes which included starchy staples, animal source protein, and vegetables.

Our findings showed that problem nutrients and dietary inadequacy were typical in diet of under-five children, even in food-secure areas. Locally available nutrient-dense foods have the potential to improve dietary adequacy in most districts for most problem nutrients, and should thus be promoted through FBRs (the nutrition specific intervention) as well as by ensuring local availability and access such as through agriculture, fishery, and market intervention (the nutrition sensitive intervention). Additional intervention(s), particularly for iron in infants, are required to ensure dietary adequacy.

## Supporting information

S1 Table Nutrient-dense foods for under-five children in the 37 districts. (XLSX)

S2 Table The 2-best diets, worst-case scenario and optimized FBRs for 6-11mo children. (XLSX)

S3 Table The 2-best diets, worst-case scenario and optimized FBRs for 12-23mo children. (XLSX)

S4 Table The 2-best diets, worst-case scenario and optimized FBRs for 24-35mo children. (XLSX)

S5 Table The 2-best diets, worst-case scenario and optimized FBRs for 36-59mo children. (XLSX)
