# Correction: Problem nutrients in diet of under-five children and district food security status: Linear programming analyses of 37 stunting priority districts in Indonesia

**DOI:** 10.1371/journal.pone.0334462

**Published:** 2025-10-13

**Authors:** Umi Fahmida, Indriya Laras Pramesthi, Sari Kusuma, Arienta R. P. Sudibya, Rahmawati Rahmawati, Dini Suciyanti, Gusnedi Gusnedi, Aly Diana

There is information missing from the Funding Statement. The correct Funding Statement is as follows:

This study was supported by grants from The Global Alliance for Improved Nutrition (GAIN) Indonesia (Contract ID:202015874; Project number: ID117C16), and the South East Asian Ministers of Education Organization Regional Centre for Food and Nutrition (SEAMEO RECFON). The funders did not play any role in the study design, data collection and analysis, decision to publish, or preparation of the manuscript.

There is also information missing from the Acknowledgements. The full statement is provided below:

We are grateful to the Directorate of Nutrition, Ministry of Health Indonesia for providing access to dietary data for LP analysis and to the academic partners who developed the draft of FBRs in LP Optifood analyses: Health Polytechnic Ministry of Health of Indonesia (Poltekkes Kemenkes RI), including Poltekkes Kemenkes Jakarta II (Dara Humaira, Ratih Puspitaningsih), Kendari (Sri Rizki), Makassar (Manjilala), Malang (Sugeng Iwan), Mataram (Irianto), Medan (Tetty H. Doloksaribu), Padang (Edmon), Pangkal Pinang (Ori P. Enardi), Pontianak (Martinus Ginting), Riau (Muharni), Semarang (Heni Hendriyani), Surabaya (Annas Buanasita), Sorong (Anjar Briliannita), Tasikmalaya (Priyo Sulistiyono), and Yogyakarta (Primiaji), as well as Universitas Airlangga (Dini R. Andrias), Universitas Brawijaya (Nia Wirawan, Ilmia Fahmi), Universitas Diponegoro (Sulistyawati, Naintina Lisnawati), and Universitas Udayana (Kadek T. Adhi).

We would also like to thank Aang Sutrisna (GAIN) and Hera Nurlita (Ministry of Health Indonesia) for their support of this study.
